# The Prevention of Consumption

**Published:** 1896-03

**Authors:** 


### The Prevention of Consumption.

Dr. B. W. Richardson, in a late number of the *Asclepiad*, states that an observation of the following rules will benefit those having a tendency toward pulmonary tuberculosis:

1. Pure air for breathing is the first rule for the prevention of consumption.
2. Active exercise, outdoor as much as possible, is essential for the prevention of consumption.
3. Uniform climate is important for consumptives.
4. The dress of the consumptive should sustain uniform warmth.
5. The hours of rest should be carefully regulated by the sunlight.
6. Outdoor occupation is preventive.
7. Amusements of consumptives should favor muscular development and sustain healthy respiration.
8. Cleanliness in the broadest sense is of special moment.
9. Every precaution should be taken to avoid colds.
10. The diet of consumptive people should be ample, with full proportion of the respiratory foods.—*Indian Lancet.*

